# Comparison of the Effects of Acarbose and TZQ-F, a New Kind of Traditional Chinese Medicine to Treat Diabetes, Chinese Healthy Volunteers

**DOI:** 10.1155/2014/308126

**Published:** 2014-04-06

**Authors:** Huang Yuhong, Fu Wenxu, Li Yanfen, Liu Yu, Li Ziqiang, Yang Liu, Liu Shirong, Sun Jinxia, Li Na, Wang Baohe, Gao Xiumei, Zhang Deqin

**Affiliations:** ^1^The Second Affiliated Hospital of Tianjin University of Traditional Chinese Medicine, No. 816 Zhenli Road, Hebei District, Tianjin 300150, China; ^2^Tasly Research Institute, Tasly Pharmaceutical Group Co., Ltd., No. 2 Pujihe East Road, Beichen District, Tianjin 300041, China; ^3^Institute of Traditional Chinese Medicine, Tianjin University of Traditional Chinese Medicine, No. 312 Anshanxi Road, Nankai District, Tianjin 300193, China; ^4^Key Laboratory of Pharmacology of Traditional Chinese Medical Formulae, Ministry of Education, No. 312 Anshanxi Road, Nankai District, Tianjin 300193, China

## Abstract

*Ethnopharmacological Relevance*. TZQ-F has been traditionally used in Traditional Chinese Medicine as a formula for the treatment of diabetes. * Aim of the Study*. This study aims to compare the pharmacologic effects and gastrointestinal adverse events between TZQ-F and acarbose. * Methods*. The double-blind randomized placebo-controlled fivefold crossover study was performed in 20 healthy male volunteers. Plasma glucose, plasma IRI, and plasma C-peptide were measured to assess the pharmacologic effects. Flatus and bowel activity were measured to assess the adverse event of gastrointestinal effect. * Results*. 3 and 4 tablets of TZQ decreased the *C*_max_ of plasma glucose compared with that of the previous day and with placebo. 3 tablets also decreased *C*_max_ of plasma C-peptide compared with placebo. 4 tablets increased *C*_max_ of plasma insulin after breakfast and the AUC of plasma C-peptide after breakfast and dinner. 2 tablets did not decrease plasma glucose and elevated the *C*_max_ and AUC of C-peptide after breakfast and dinner, respectively. Acarbose 50 mg decreased the *C*_max_ of plasma insulin and C-peptide after breakfast and the *C*_max_ of plasma glucose and C-peptide after dinner. The subjects who received TZQ did not report any abdominal adverse events. * Conclusions*. 3 tablets of TZQ have the same effects as the acarbose.

## 1. Introduction

The prevalence of type 2 diabetes is rising exponentially and it has become a global health priority [1]. The International Diabetes Federation has predicted that the number of individuals with diabetes is likely to increase from 382 million in 2013 to 592 million in 2035 [2]. All types of diabetes mellitus are characterized by an increased cardiovascular risk, which is mostly pronounced in type 2 diabetes. This has a special significance, as this most common type of the disease develops asymptomatically in the majority of the cases, and therefore the detection of type 2 diabetes is often delayed and the advanced complications are frequently presented at the time of the diagnosis.

Impaired glucose tolerance (IGT), as well as insulin resistance, is known to be associated with an increased risk of type 2 diabetes and hypertension, which are well-recognized risk factors for cardiovascular diseases [3–5]. Considering the heavy burden of these metabolic disorders on the public health, improvement of IGT and/or insulin resistance is a supremely important health issue.

Traditional Chinese Medicine (TCM) has been used in treating diabetes mellitus for almost twenty centuries in China. TangZhiQing Formula (TZQ-F) is a well-known antidiabetic formula containing five herbs, which are* Paeonia lactiflora *Pall., root,* Morus alba *L., leaf,* Nelumbo nucifera *Gaertn., leaf,* Salvia miltiorrhiza bge*., roots, and* Crataegus pinnatifida bge*., leaf. TZQ-F comes from a prescription named* Salvia miltiorrhiza powder*, which was recorded in* Taiping Holy Prescriptions for Universal Relief* of Song dynasty of China. The results of antidiabetic studies showed TZQ-F can reduce blood glucose, total cholesterol, and triglyceride levels of KK-Ay mice after 4 weeks of oral administration [6].

*α*-Glucosidase inhibitors are commonly used for type 2 diabetes mellitus. *α*-Glucosidase inhibitors reduce the absorption of carbohydrates from the small intestine and thereby lower the levels of postprandial blood glucose. Plants and microorganisms are rich sources of *α*-glucosidase inhibitors. Screening of *α*-glucosidase inhibitors from plants and synthetic sources has been a hot research topic [7]. Our previous study [8] showed that TZQ-F possesses blood glucose lowering effects, possibly by inhibiting intestinal *α*-glucosidase. As a continuing study, this paper compares pharmacologic effects and gastrointestinal adverse events associated with TZQ-F and acarbose which is an *α*-glucosidase inhibitor being marketed for 30 years approximately.

## 2. Patients and Methods

The subjects were 20 male volunteers aged from 19 to 29 years (mean ± SD, 23.35 ± 2.62 years), who were in good health, as determined by history, physical examination, and routine laboratory investigations. Body mass index was 18.94 to 23.94 kg/m^2^ (mean ± SD, 21.47 ± 1.59 kg/m^2^). Informed written consents were given before the trial began, and the participants were free to withdraw at any time during the study. One subject withdrew after period 3, because he has to go back homeland to take care of his ill mother.

The drug TZQ-F and placebo were produced by ShanDong Buchang Shenzhou Pharmaceutical Co., Ltd., which was approved to produce tablets in November 2010 by CFDA (China Food and Drug Administration). In May 2012, the trial protocol was approved by the Ethics Committees of the Second Affiliated Hospital of Tianjin University of Traditional Chinese Medicine where the study was conducted. The registration number from the international clinical trial net is ChiCTR-TTRCC-12002866.

The subjects were hospitalized from the night before the first day of the study (on which no drugs were given) until the morning after the second day of the study (on which the drugs were administered). During hospitalization, only prescribed meals were allowed; meals were eaten at 8 am, 12 pm, and 6 pm. The same three meals were served on the first and second day of the study. Carbohydrate was supplied as bread at breakfast and rice at lunch and dinner. Plasma glucose, immunoreactive insulin (IRI), and C-peptide levels were monitored at breakfast and dinner. Energy available in breakfast, lunch, and dinner was 691 kcal (carbohydrate, 104 g; fat, 19 g; protein, 26 g), 922 kcal (carbohydrate, 138 g; fat, 26 g; protein, 34 g), and 687 kcal (carbohydrate, 103 g; fat, 19 g; protein, 26 g), respectively. Caffeinated and alcoholic drinks were prohibited during hospitalization.

Subjects were prohibited from vigorous exercise during the study. No drug except the test drugs was administered from after the screening test (1 month before the study) until the end of the fifth treatment period of the study.

### 2.1. Study Design

The study was conducted according to a randomized, double-blind, placebo-controlled, fivefold, crossover design. No drugs were given on the first day, and the following drugs were administered on the second day: acarbose, 2 tablets, 3 tablets, and 4 tablets of TZQ, or placebo 3 times a day. The specification of TZQ is 0.64 g per tablet. See Table 1 of the dosage regimen of the five groups.

**Figure 1:**
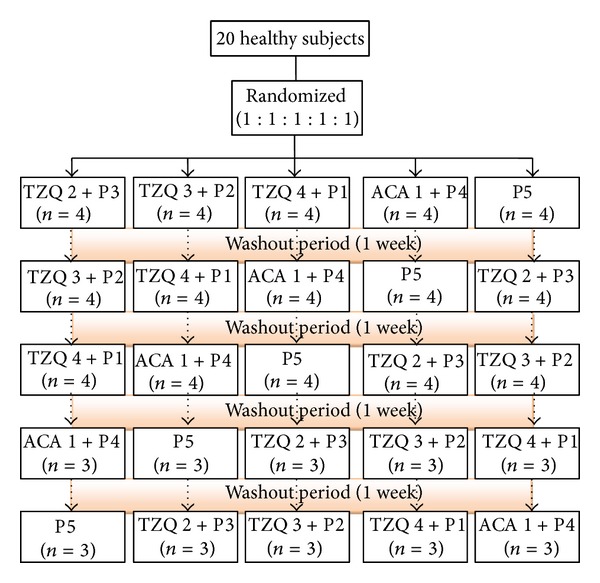
Flow of study subjects. ACA1 denotes 1 tablet of acarbose; TZQ 1–TZQ 5 denotes 1–5 tablets of TZQ; P1 denotes 1 tablet of acarbose simulation agent; P2 denotes 1 tablet of acarbose simulation agent and 1 tablet of TZQ simulation agent; P3 denotes 1 tablet of acarbose simulation agent and 2 tablets of TZQ simulation agent; P4 denotes 1 tablet of acarbose simulation agent and 3 tablets of TZQ simulation agent; P5 denotes 1 tablet of acarbose simulation agent and 4 tablets of TZQ simulation agent.

The subjects were divided into five groups; each group contains four subjects using a balanced Latin square of four subjects by five kinds of treatments. The drugs were administered with 200 mL water just before each meal during the five treatment periods. The drug-free washout period between each two treatment periods was 1 week (Figure 1).

To investigate pharmacologic effects, plasma glucose, IRI, and C-peptide levels were determined before and 0.25, 0.5, 1, 1.5, 2, and 3 hours after the breakfast and dinner. Area under the plasma concentration-time curve (AUC) for plasma glucose, IRI, and C-peptide was calculated using the trapezoidal rule.

To investigate the gastrointestinal effects, subjective symptoms, flatus, and bowel activity were monitored. The severity, time of onset, and time of disappearance of all symptoms of the subjects were recorded on the designated form. The frequency and severity of flatus as mild, moderate, or serious were also recorded. The flatus score was calculated by multiplying the frequency by 3 points for serious flatus, 2 for moderate flatus, and 1 for mild flatus. For assessment of bowel activity, the frequency of defection was recorded and the stools were photographed. The stools were then classified from the photographs as watery, loose, soft, firm, or hard bolus. Stool scores were calculated by multiplying the frequency by a score ranging from 5 points for watery stool to 1 point for hard bolus.

### 2.2. Analytic Method

IRI and C-peptide level were determined by chemiluminescence (ADVIA Centaur, Siemens). Plasma glucose level was determined by the glucose oxidase method.

### 2.3. Statistical Analysis

Analysis of variance (ANOVA) was used to test the effects of treatment on maximum concentration (*C*_max⁡_) and AUC for the change of plasma glucose, IRI, and C-peptide. If the drug effect was found to be significant by ANOVA, paired *t*-test was used to test the effects of each group before and after the treatment; multiple comparison of LSD was used to test the difference between the treatment and the placebo. For analysis of flatus and bowel activity, the one-sample Wilcoxon test was used. All tests were two-tailed, and the level of significance was set at 0.05.

## 3. Results

### 3.1. Plasma Glucose

The drug treatments significantly decreased *C*_max⁡_ of the plasma glucose after dinner (*P* = 0.0003). Compared with placebo, reduction in *C*_max⁡_ of plasma glucose was significant in acarbose 50 mg, 3 tablets and 4 tablets of TZQ after dinner, respectively (Figure 3, Table 2). Compared with before treatment, acarbose 50 mg, 3 tablets and 4 tablets of TZQ also showed statistically significant role in decreasing *C*_max⁡_ of plasma glucose after dinner (Figure 3, Table 2). All of the drug treatments did not change plasma glucose after the breakfast significantly.

### 3.2. Plasma Insulin

**Figure 2:**
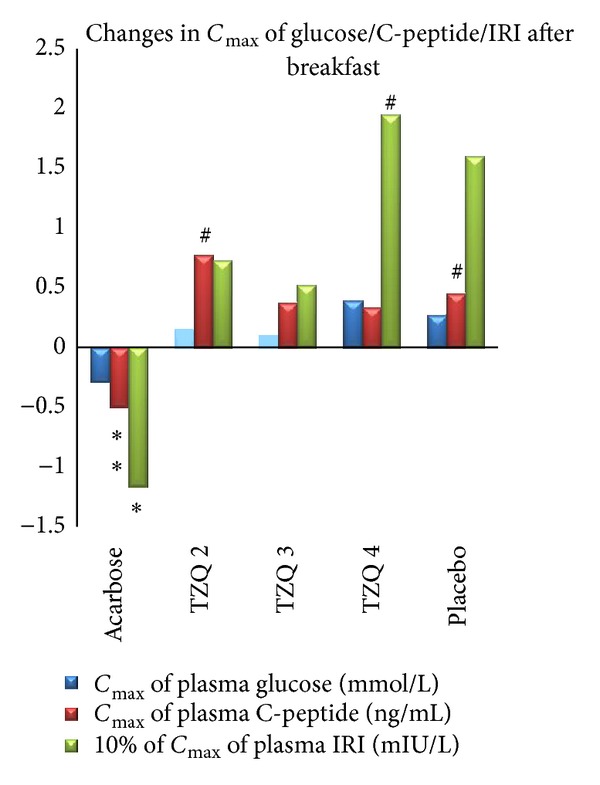
Mean change in *C*_max⁡_ of glucose/C-peptide/IRI after breakfast from first day (no drug administration) to second day (drug administration). Reduction in plasma C-peptide and IRI was significant in acarbose group (*P < 0.05 versus placebo; ^#^ *P* < 0.05 versus before treatment). Elevation of *C*_max⁡_ of plasma IRI was significant in TZQ 4 group (^#^ *P* < 0.05 versus before treatment). Elevation of *C*_max⁡_ of plasma C-peptide was significant in TZQ 2 and placebo groups (^#^ *P* < 0.05 versus before treatment).

Plasma insulin changed significantly after the treatment. The acarbose 50 mg decreased *C*_max⁡_ of plasma insulin after breakfast and dinner, respectively. 3 tablets of TZQ decreased *C*_max⁡_ of plasma insulin after dinner only. Compared with placebo, acarbose significantly decreased the *C*_max⁡_ of plasma of IRI after breakfast (Figure 2, Table 3). 4 tablets of TZQ increased *C*_max⁡_ of plasma insulin after breakfast (*P* = 0.015 versus before treatment) (Figure 2, Table 3).

### 3.3. C-Peptide

**Figure 3:**
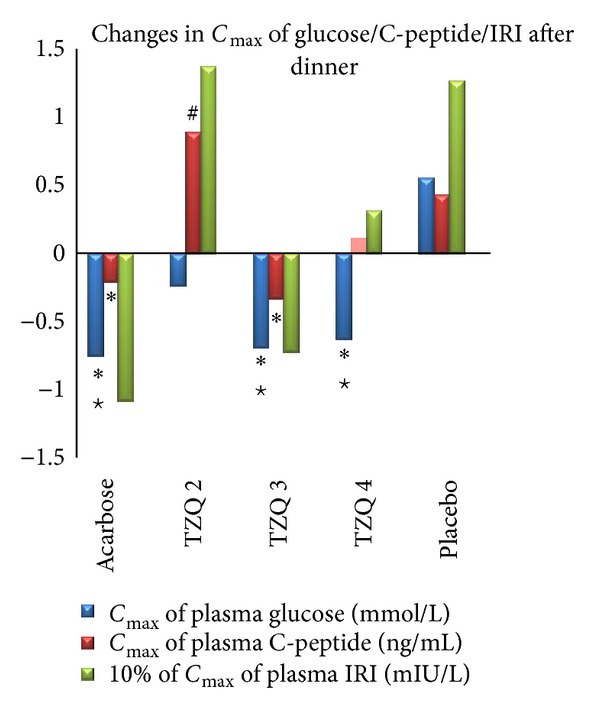
Mean change in *C*_max⁡_ of glucose/C-peptide/IRI after dinner from first day (no drug administration) to second day (drug administration). Reduction in *C*_max⁡_ of plasma glucose was significant in acarbose and TZQ 3 and TZQ 4 groups (*P < 0.05 versus placebo; ^#^ *P* < 0.05 versus before treatment). Reduction in *C*_max⁡_ of plasma C-peptide was significant in acarbose and TZQ 3 groups (*P < 0.05 versus placebo; ^#^ *P* < 0.05 versus before treatment). Elevation of *C*_max⁡_ of plasma C-peptide was significant in TZQ 2 group (^#^ *P* < 0.05 versus before treatment).

Plasma C-peptide changed significantly after the treatment. Compared with placebo, acarbose 50 mg significantly decreased *C*_max⁡_ of plasma C-peptide after breakfast and dinner, respectively (Figures 2 and 3, Table 4). 3 tablets of TZQ significantly decreased *C*_max⁡_ of plasma C-peptide only after dinner (Figure 3, Table 4).

**Figure 4:**
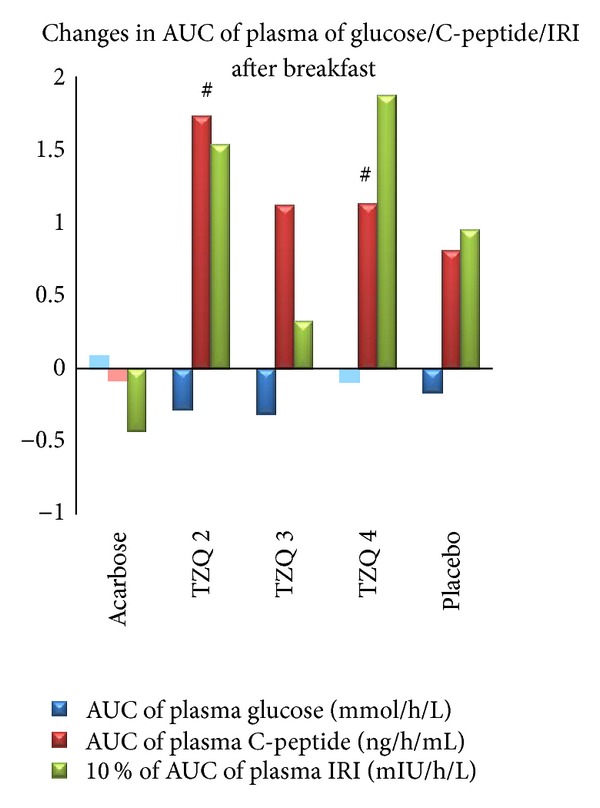
Mean change in AUC of glucose/C-peptide/IRI after breakfast from first day (no drug administration) to second day (drug administration). Elevation in AUC of plasma C-peptide was significant in TZQ 2 and TZQ 4 group (^#^ *P* < 0.05 versus before treatment).

**Figure 5:**
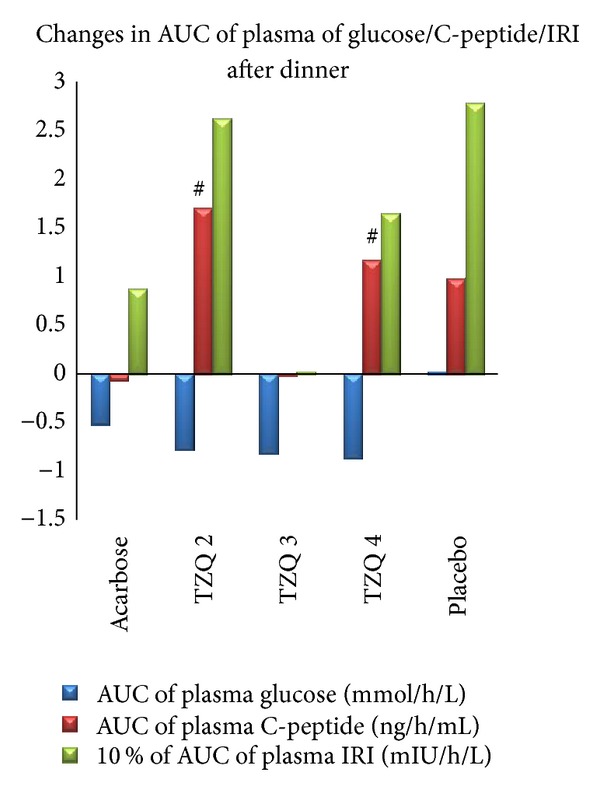
Mean change in AUC of glucose/C-peptide/IRI after dinner from first day (no drug administration) to second day (drug administration). Elevation of AUC of plasma C-peptide was significant in TZQ 2 and TZQ 4 groups (^#^ *P* < 0.05 versus before treatment).

Compared with before treatment, elevation of *C*_max⁡_ of plasma C-peptide after breakfast was significant in 2 tablets of TZQ and placebo (Figure 2, Table 4). 2 tablets of TZQ significantly elevated *C*_max⁡_ of plasma C-peptide after dinner (Figure 3, Table 4). Besides, 2 tablets of TZQ and 4 tablets of TZQ significantly elevated the AUC of plasma C-peptide after breakfast and dinner, respectively (Figures 4 and 5, Table 4).

### 3.4. Gastrointestinal Effects

**Figure 6:**
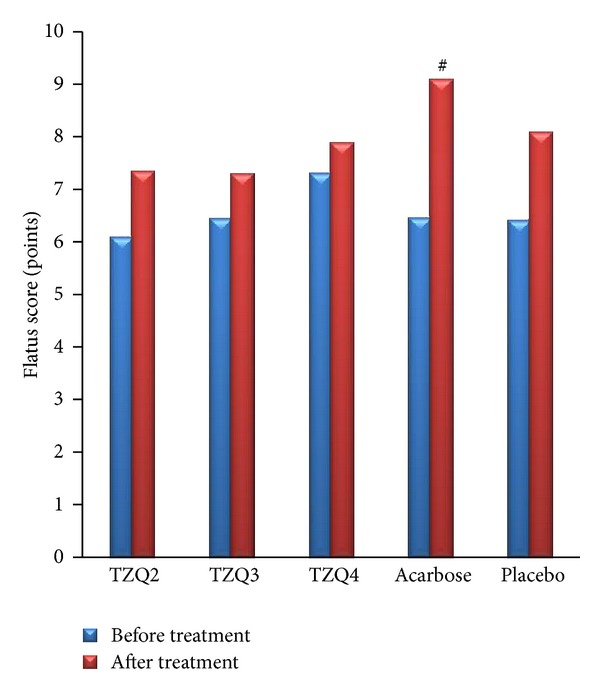
Mean flatus score before (blue column) and during (red column) administration of 2 tablets, 3 tablets, and 4 tablets of TZQ, acarbose, and placebo on 19 volunteers. Mean flatus score was significantly elevated in acarbose dose (^#^ *P* < 0.05 versus first day (before treatment)).

Flatus scores did not increase significantly during the treatment compared with that of the previous day in subjects receiving TZQ and placebo but increased significantly in acarbose 50 mg group (*P* = 0.036). There were no significant differences in flatus scores between groups (Figure 6).

**Figure 7:**
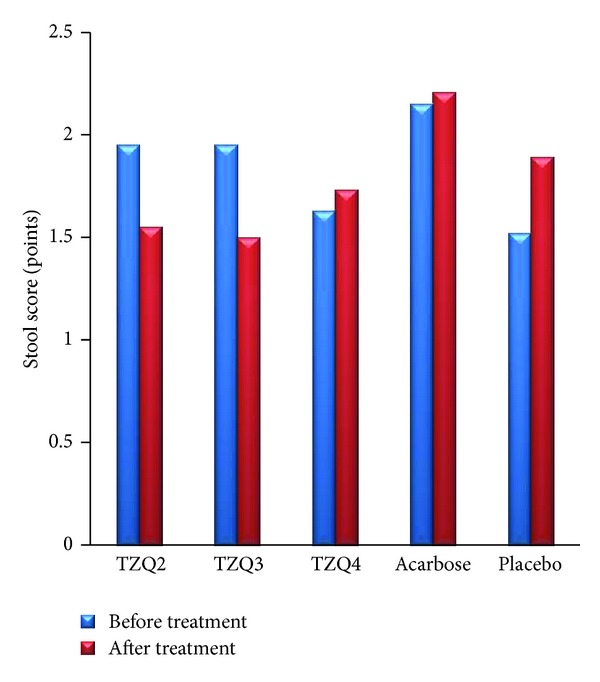
Mean stool score before (blue column) and during (red column) administration of 2 tablets, 3 tablets, and 4 tablets of TZQ, acarbose, and placebo on 19 volunteers.

Stool scores did not increase significantly during the treatment compared with that of the previous day in all groups (Figure 7).

## 4. Discussion and Conclusions

Previous* in vitro* mechanism study of TZQ showed that three fractions of TZQ had strong inhibition effects on rat intestinal disaccharase, which are mulberry leaf total alkaloids fraction, mulberry leaf total flavonoid fraction, and hawthorn leaf total flavonoids fraction. Particularly, the mulberry leaf total alkaloids fraction (IC50 = 0.26 *μ*g/mL for sucrase and 0.05 *μ*g/mL for maltase) is stronger than the positive control of acarbose [8]. So in the clinical practice, TZQ may affect the plasma glucose at the similar style of acarbose.

3 tablets and 4 tablets of TZQ have significantly decreased the *C*_max⁡_ of the plasma glucose compared with that of previous day and with placebo. Like acarbose, 3 tablets of TZQ also decreased *C*_max⁡_ of plasma C-peptide compared with placebo. 4 tablets of TZQ significantly increased *C*_max⁡_ of plasma insulin after breakfast and the AUC of plasma C-peptide after breakfast and dinner. Though 2 tablets of TZQ did not decrease plasma glucose significantly, it elevated the *C*_max⁡_ and AUC of C-peptide after breakfast and dinner, respectively. Acarbose 50 mg decreased the *C*_max⁡_ of plasma insulin and C-peptide after breakfast and the *C*_max⁡_ of plasma glucose and C-peptide after dinner significantly. It shows that the 3 tablets of TZQ have the same effects as the acarbose which is to inhibit the postprandial increase in blood glucose levels by inhibiting and delaying digestion and absorption of carbohydrate.

3 tablets of TZQ and the alpha-glucosidase inhibitor, acarbose, inhibited the postprandial increase in plasma glucose levels and decreased insulin secretion to maintain normoglycemia in nondiabetic subjects. Inhibition of the postprandial increase in plasma glucose was more marked at dinner than at breakfast. This was partially due to cumulative effect of alpha-glucosidase inhibitors [9]. Although data are not available in humans, the turnover time of disaccharidases in the rat has been reported to be 11.5 hours. Thus it would appear reasonable that because TZQ or acarbose was given at every meal, the cumulative effects would be observed at dinner.

TZQ 2-tablet dose increased the *C*_max⁡_ and AUC of plasma C-peptide after breakfast and dinner, respectively, and TZQ 4-tablet dose significantly increased the AUC of plasma C-peptide after breakfast and dinner, respectively, and thus the TZQ 2- and TZQ 4-tablet dose possibly increased the insulin secretion. Traditionally, Chinese herbs are used as a formulated decoction, a specific combination of different herbs, prepared using a unique methodology to achieve a specific efficacy. The herbs in the formula are not simply added together in a cumulative fashion. Instead, they are precisely combined according to a particular principle. The characteristics of Chinese herbal medicine include multiple-component and multitarget actions [10]. TZQ is one of these typical Chinese herbal formulas that different dose may produce different effect.

The incidence of abdominal adverse events has previously been reported with acarbose. Our study also demonstrated the same result. The subjects receiving TZQ did not report abdominal adverse events. That may be because of the characteristics of Chinese herbal medicine, that is, multiple-component and multitarget actions. It will improve the patient's compliance.

Although the *C*_max⁡_ and AUC of plasma glucose after dinner decreased significantly with acarbose and TZQ, the reduction rate was small. As the subjects were not diabetic, postprandial plasma glucose levels were maintained within a narrow range, resulting in a small reduction in plasma glucose levels when a normal amount of food was ingested. TZQ has been used for many centuries in China to treat diabetes, but the clinical evidence has not been established. Based on this study, a multicenter clinical trial will be carried out by our team to evaluate the effect of TZQ on the diabetes mellitus in the near future.

## Figures and Tables

**Figure 8:**
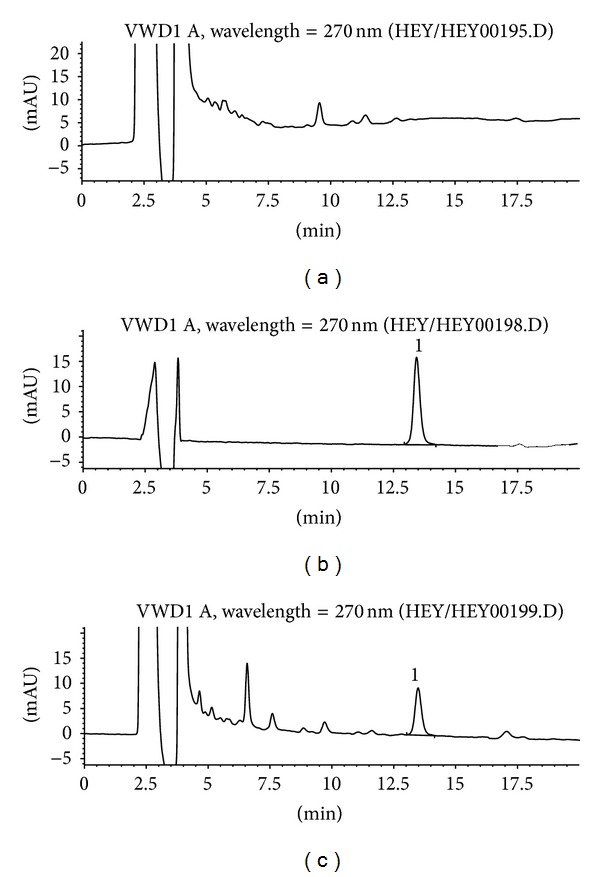
Typical HPLC chromatograms of marker compound nuciferine in TZQ: (a) blank control; (b) standard compound control; (c) TZQ sample. 1, nuciferine from* Nelumbo nucifera* Gaertn., leaf.

**Figure 9:**
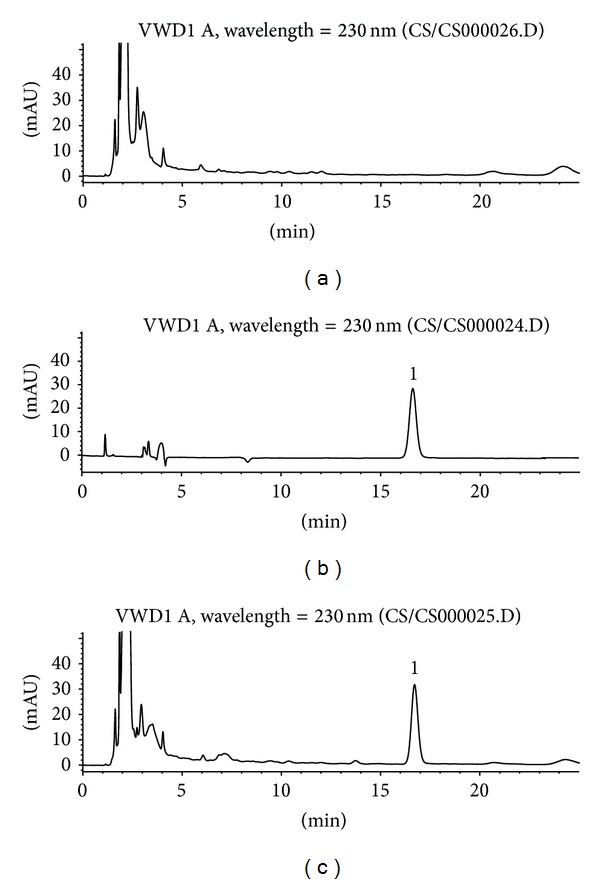
Typical HPLC chromatograms of marker compound paeoniflorin in TZQ: (a) blank control; (b) standard compound control; (c) TZQ sample. 1, paeoniflorin from* Paeonia lactiflora* Pall., root.

**Table 1:** Dosage regimens of five groups.

Groups	Acarbose	TZQ			
Acarbose	●	□	□	□	□
TZQ 2 tablets	○	■	■	□	□
TZQ 3 tablets	○	■	■	■	□
TZQ 4 tablets	○	■	■	■	■
Placebo	○	□	□	□	□

“●” means one 50 mg tablet of acarbose; “○” means one 50 mg tablet of acarbose simulation agent; “■” means one 0.64 g tablet of TZQ; “□” means one 0.64 g tablet of TZQ simulation agent.

**Table 2:** Maximum concentration (*C*_max⁡_) and area under the plasma concentration-time curve (AUC) of plasma glucose after breakfast and dinner on 19 healthy volunteers (mean ± SEM).

	Dose (mg/d, p.o.)	*C*_max⁡_ of plasma glucose (mmol/L) after breakfast			
		*n*	First day	Second day	Change
Acarbose	50	19	6.93 ± 0.84	6.64 ± 0.86	−0.29 ± 0.74
TZQ- 2 tables	1280	19	6.75 ± 0.74	6.90 ± 1.06	0.15 ± 0.95
TZQ- 3 tables	1920	19	6.83 ± 1.03	6.92 ± 0.73	0.10 ± 0.84
TZQ- 4 tables	2560	19	6.78 ± 0.99	7.17 ± 0.92	0.39 ± 0.82
Placebo	—	19	6.97 ± 1.14	7.23 ± 0.95	0.27 ± 0.84

	Dose (mg/d, p.o.)	*C*_max⁡_ of plasma glucose (mmol/L) after dinner			
		*n*	First day	Second day	Change^#^

Acarbose	50	19	7.28 ± 1.03	6.54 ± 0.89*	−0.75 ± 0.88
TZQ- 2 tables	1280	19	7.38 ± 0.84	7.14 ± 1.38	−0.24 ± 1.40
TZQ- 3 tables	1920	19	7.69 ± 0.91	7.00 ± 1.08*	−0.69 ± 0.90
TZQ- 4 tables	2560	19	7.35 ± 0.97	6.72 ± 0.77*	−0.63 ± 1.09
Placebo	—	19	7.06 ± 0.86	7.60 ± 1.53	0.55 ± 1.32

	Dose (mg/d, p.o.)	AUC of Plasma glucose (mmol·h/L) after breakfast			
		*n*	First day	Second day	Change

Acarbose	50	19	16.29 ± 2.01	16.38 ± 1.79	0.09 ± 1.45
TZQ- 2 tables	1280	19	16.60 ± 1.43	16.31 ± 1.54	−0.28 ± 1.19
TZQ- 3 tables	1920	19	16.65 ± 1.91	16.34 ± 1.08	−0.31 ± 1.50
TZQ- 4 tables	2560	19	16.79 ± 2.01	16.69 ± 1.77	−0.09 ± 1.44
Placebo	—	19	16.55 ± 1.69	16.39 ± 1.83	−0.16 ± 1.04

	Dose (mg/d, p.o.)	AUC of plasma glucose (mmol·h/L) after dinner			
		*n*	First day	Second day	Change

Acarbose	50	19	17.59 ± 1.99	17.06 ± 2.03	−0.52 ± 1.78
TZQ- 2 tables	1280	19	18.01 ± 0.41	17.23 ± 3.32	−0.78 ± 3.43
TZQ- 3 tables	1920	19	18.08 ± 1.87	17.25 ± 2.91	−0.82 ± 2.24
TZQ- 4 tables	2560	19	17.98 ± 1.63	17.10 ± 2.16	−0.87 ± 2.27
Placebo	—	19	17.29 ± 1.60	17.32 ± 2.84	0.03 ± 2.79

*P < 0.05 versus first day (before treatment). ^#^ *P* < 0.05 five treatments compared using ANOVA.

**Table 3:** Maximum concentration (*C*_max⁡_) and area under the plasma concentration-time curve (AUC) of plasma IRI after breakfast and dinner on 19 healthy volunteers (mean ± SEM).

	Dose (mg/d, p.o.)	*C*_max⁡_ of plasma IRI (mIU/L) after breakfast			
		*n*	First day	Second day	Change^#^
Acarbose	50	19	75.77 ± 50.30	64.10 ± 35.92	−11.67 ± 32.69
TZQ- 2 tables	1280	19	80.02 ± 51.86	86.83 ± 49.25	7.28 ± 29.33
TZQ- 3 tables	1920	19	85.65 ± 49.82	90.83 ± 44.86	5.18 ± 24.35
TZQ- 4 tables	2560	19	77.42 ± 40.10	96.85 ± 49.99*	19.43 ± 31.44
Placebo	—	19	85.36 ± 46.86	101.32 ± 54.16	15.96 ± 39.25

	Dose (mg/d, p.o.)	*C*_max⁡_ of plasma IRI (mIU/L) after dinner			
		*n*	First day	Second day	Change^#^

Acarbose	50	19	54.85 ± 37.42	44.00 ± 30.64	−10.85 ± 35.69
TZQ- 2 tables	1280	19	42.78 ± 24.93	56.50 ± 29.19	13.72 ± 23.77
TZQ- 3 tables	1920	19	55.79 ± 29.29	48.25 ± 27.37	−7.27 ± 30.70
TZQ- 4 tables	2560	19	48.86 ± 30.92	51.99 ± 28.55	3.13 ± 20.49
Placebo	—	19	52.43 ± 26.17	65.09 ± 3.62	12.66 ± 36.66

	Dose (mg/d, p.o.)	AUC of plasma IRI (mIU/h/L) after breakfast			
		*n*	First day	Second day	Change

Acarbose	50	19	107.73 ± 70.16	103.45 ± 50.89	−4.28 ± 39.61
TZQ- 2 tables	1280	19	113.90 ± 61.00	129.24 ± 60.36	15.34 ± 37.52
TZQ- 3 tables	1920	19	127.50 ± 70.60	130.74 ± 61.47	3.24 ± 43.54
TZQ- 4 tables	2560	19	122.72 ± 71.76	141.48 ± 68.06	18.76 ± 39.57
Placebo	—	19	128.43 ± 79.93	137.97 ± 63.00	9.54 ± 47.88

	Dose (mg/d, p.o.)	AUC of plasma IRI (mIU/h/L) after dinner			
		*n*	First day	Second day	Change

Acarbose	50	19	66.01 ± 35.68	74.75 ± 43.69	8.73 ± 39.39
TZQ- 2 tables	1280	19	66.45 ± 31.29	92.68 ± 36.51	26.23 ± 28.88
TZQ- 3 tables	1920	19	84.95 ± 49.02	85.23 ± 43.16	0.28 ± 47.98
TZQ- 4 tables	2560	19	77.84 ± 45.67	94.32 ± 47.68	16.48 ± 35.71
Placebo	—	19	68.39 ± 29.40	96.21 ± 7.93	27.82 ± 26.31

*P < 0.05 versus first day (before treatment). ^#^ *P* < 0.05 five treatments compared using ANOVA.

**Table 4:** Maximum concentration (*C*_max⁡_) and area under the plasma concentration-time curve (AUC) of plasma C-peptide after breakfast and dinner on 19 healthy volunteers (mean ± SEM).

	Dose (mg/d, p.o.)	*C*_max⁡_ of plasma C-peptide (ng/mL) after breakfast			
		*n*	First day	Second day	Change^#^
Acarbose	50	19	5.13 ± 2.20	4.63 ± 1.29*	−0.50 ± 1.19
TZQ- 2 tables	1280	19	4.68 ± 1.71	5.46 ± 1.98*	0.77 ± 1.09
TZQ- 3 tables	1920	19	5.20 ± 2.33	5.57 ± 2.11	0.37 ± 1.69
TZQ- 4 tables	2560	19	5.08 ± 2.17	5.41 ± 2.04	0.33 ± 1.11
Placebo	—	19	5.09 ± 2.11	5.54 ± 2.29*	0.45 ± 1.46

	Dose (mg/d, p.o.)	*C*_max⁡_ of plasma C-peptide (ng/mL) after dinner			
		*n*	First day	Second day	Change^#^

Acarbose	50	19	4.56 ± 1.49	4.35 ± 1.80	−0.21 ± 1.20
TZQ- 2 tables	1280	19	4.31 ± 1.17	5.20 ± 1.56*	0.89 ± 0.76
TZQ- 3 tables	1920	19	4.98 ± 1.58	4.64 ± 1.50	−0.33 ± 1.00
TZQ- 4 tables	2560	19	4.50 ± 1.80	4.61 ± 1.45	0.11 ± 1.17
Placebo	—	19	4.70 ± 1.49	5.13 ± 1.57	0.43 ± 1.15

	Dose (mg/d, p.o.)	AUC of plasma C-peptide (ng/h/mL) after breakfast			
		*n*	First day	Second day	Change^#^

Acarbose	50	19	9.83 ± 3.40	9.76 ± 2.92	−0.08 ± 1.23
TZQ- 2 tables	1280	19	9.67 ± 3.04	11.40 ± 3.74*	1.73 ± 1.72
TZQ- 3 tables	1920	19	10.26 ± 4.26	11.39 ± 3.90	1.12 ± 2.45
TZQ- 4 tables	2560	19	10.32 ± 4.37	11.45 ± 4.33*	1.13 ± 2.07
Placebo	—	19	10.40 ± 4.16	11.22 ± 4.22	0.81 ± 1.78

	Dose (mg/d, p.o.)	AUC of plasma C-peptide (ng/h/mL) after dinner			
		*n*	First day	Second day	Change^#^

Acarbose	50	19	9.29 ± 2.32	9.23 ± 2.90	−0.07 ± 1.73
TZQ- 2 tables	1280	19	9.92 ± 2.41	11.62 ± 3.08*	1.70 ± 1.42
TZQ- 3 tables	1920	19	10.66 ± 3.65	10.64 ± 3.41	−0.02 ± 2.06
TZQ- 4 tables	2560	19	9.74 ± 2.96	10.91 ± 3.53*	1.17 ± 1.94
Placebo	—	19	10.30 ± 3.14	11.28 ± 2.82	0.98 ± 2.08

*P < 0.05 versus first day (before treatment). ^#^ *P* < 0.05 five treatments compared using ANOVA.

## Appendices

### A. Quality Control of TZQ-F

The traditional Chinese herbal medicine preparation TZQ-F is a combination of five herbal ingredients—*Paeonia lactiflora *Pall., root,* Morus alba *L., leaf,* Nelumbo nucifera *Gaertn., leaf,* Salvia miltiorrhiza Bge.*, root,* Crataegus pinnatifida *Bge., leaf—manufactured under the Code of Good Manufacturing Practice by Shandong Buchang Shenzhou Pharmaceutical Co., Ltd. [8]. The TZQ tablets employed in this research were batch 120606. All the test and quality control (QC) of this product act in accordance with the “Chinese Pharmacopoeia” (2010 version). Accordingly, the marker compounds of* Nelumbo nucifera *Gaertn.,* leaf*, and* Paeonia lactiflora *Pall.,* root*, arenuciferine and paeoniflorin, respectively. A TZQ tablet contains not less than 0.33 mg of nuciferine (C_19_H_21_NO_2_) and not less than 6.2 mg of paeoniflorin (C_23_H_28_O_11_). Phenotypic trait—products are film coated tablets with a faint characteristic odour and a slightly bitter taste. The inner surface is brown after removing the coating layer. Identification—thin layer chromatographic identification test is employed to identify the five herbal ingredients. Checkup—disintegration time limited is not more than 1 hour; mass discrepancy is within the limits of 5%; microbial limit should also meet the specification.

### B. The Quantification of Nuciferine in the Tablets of TZQ

The leaf of* Nelumbo nucifera *Gaertn. is a Traditional Chinese Medicine for losing weight and has been commonly used for clearing heat, removing heatstroke, cooling blood, and stanching blood. The major phytochemicals present in lotus leaf are three aporphine alkaloids,* N*-nornuciferine,* O*-nornuciferine, and nuciferine. According to the guiding principles of the “Chinese Pharmacopoeia” (2010 version), nuciferine is regarded as a QC compound to conduct the determination of folium nelumbinis.

#### B.1. High Performance Liquid Chromatography Analysis

HPLC analyses were performed using an Agilent HPLC system (Agilent 1100 Series, Agilent Technologies, CA, USA) composed of a column heater, a sample manager, a binary solvent manager, and a variable wavelength detector. The liquid chromatograph is equipped with a 4.6 mm × 250 mm column that contains 5 *μ*m packing C18 (ZORBAX 300 Å Extend-C18, Agilent, CA, USA). The employed detection wavelength is 270 nm for nuciferine. A filtered and degassed mixture of acetonitrile, water, triethylamine, and acetic acid (33 : 64.8 : 1.5 : 0.7) is prepared. The flow rate is about 1.0 mL per minute. The column temperature is maintained at 25°C. Chromatograph the standard preparation and record the peak responses as directed for procedure: the column efficiency is not fewer than 2000 theoretical plates; the tailing factor is not more than 2.0; and the relative standard deviation for replicate injections is not more than 2.0% [11].

#### B.2. Sample Preparation

Weigh and finely powder not fewer than 10 tablets. Transfer an accurately weighed portion 1.0 g of the powder to a 100 mL volumetric flask, add 80 mL of methanol, and sonicate for about 10 minutes with intermittent shaking. Shake the flask on a mechanical shaker for about 30 minutes. Dilute with mobile phase to volume and mix. Pass a portion of this solution through a polytetrafluoroethylene membrane filter having a 0.45 *μ*m porosity, discarding the first few mL.

#### B.3. Analytical Results

Based on the analytical results, the content of marker compounds was 0.84 mg/g for nuciferine in the TZQ tablets. The analytical chromatograms are shown in Figure 8.

### C. The Quantification of Paeoniflorin in the Tablets of TZQ

The dried peeled root of* Paeonia lactiflora* Pall. is one of the Chinese traditional tonic crude drugs. Paeoniflorin, a water soluble substance isolated from the root of* P. lactiflora*, is one of the bioactive components and has been reported to exhibit anticoagulant, neuromuscular blocking, cognition-enhancing, immunoregulating, and antihyperglycemic effects. Therefore, paeoniflorin is chosen as a second QC marker compound of TZQ tablet.

#### C.1. HPLC Analysis

HPLC analyses were performed using an Agilent HPLC system (Agilent 1100 Series, Agilent Technologies, CA, USA) composed of a column heater, a sample manager, a binary solvent manager, and a variable wavelength detector. The liquid chromatograph is equipped with a 4.6 mm × 250 mm column that contains 5 *μ*m packing C18 (ZORBAX 300 Å Extend-C18, Agilent, CA, USA). The employed detection wavelength is 230 nm for paeoniflorin. A filtered and degassed mixture of acetonitrile and water (14 : 86) is prepared. The flow rate is about 1.0 mL per minute. The column temperature is maintained at 25°C. Chromatograph the standard preparation and record the peak responses as directed for procedure: the column efficiency is not fewer than 3000 theoretical plates; the tailing factor is not more than 2.0; and the relative standard deviation for replicate injections is not more than 2.0% [12].

#### C.2. Sample Preparation

Weigh and finely powder not fewer than 10 tablets. Transfer an accurately weighed portion 1.0 g of the powder to a 100 mL volumetric flask, add 80 mL of water, and sonicate for about 5 minutes with intermittent shaking. Shake the flask on a mechanical shaker for about 30 minutes. Dilute with mobile phase to volume and mix. Pass a portion of this solution through a polytetrafluoroethylene membrane filter having a 0.45 *μ*m porosity, discarding the first few mL.

#### C.3. Analytical Results

Based on the analytical results, the content of marker compounds was 9.69 mg/g for paeoniflorin in the TZQ tablets. The analytical chromatograms are shown in Figure 9.
